# Inviscid and Viscous Interactions in Subsonic Corner Flows

**DOI:** 10.1155/2013/940862

**Published:** 2013-06-27

**Authors:** Kung-Ming Chung, Po-Hsiung Chang, Keh-Chin Chang

**Affiliations:** ^1^Aerospace Science and Technology Research Centre, National Cheng Kung University, Tainan 711, Taiwan; ^2^Institute of Aeronautics and Astronautics, National Cheng Kung University, Tainan 711, Taiwan

## Abstract

A flap can be used as a high-lift device, in which a downward deflection results in a gain in lift at a given geometric angle of attack. To characterize the aerodynamic performance of a deflected surface in compressible flows, the present study examines a naturally developed turbulent boundary layer past the convex and concave corners. This investigation involves the analysis of mean and fluctuating pressure distributions. The results obtained indicate strong inviscid-viscous interactions. There are upstream expansion and downstream compression for the convex-corner flows, while the opposite trend is observed for the concave-corner flows. A combined flow similarity parameter, based on the small perturbation theory, is proposed to scale the flow characteristics in both subsonic convex- and concave-corner flows.

## 1. Introduction

Corner flows occur in a wide variety of internal and external aerodynamic problems. Previous studies have been mainly on supersonic and hypersonic speeds [1–4]. In subsonic flow regime, aircraft designs have employed flaps for take-off and landing performance and ailerons for routine turning maneuver. A study by Bolonkin and Gilyard [5] demonstrated that active modification of control surfaces (variable camber wings) potentially could play a role in performance optimization for fighter aircraft and transport aircraft. While cruising, there could be more than 10 percent in maximizing the lift-to-drag ratio, especially for nonstandard flight conditions [6]. Further, a simplified model of a deflected surface comprised convex-corner and concave-corner flows. For a compressible convex-corner flow (or upper deflected surface), there are strong upstream expansion and downstream compression, caused by viscous-inviscid interactions, near the corner. The displacement thickness near the corner is affected by the overlapping region that lies between the viscous sublayer and the main part of the boundary layer [7]. On the lower deflected surface (or concave corner), the flow decelerates upstream of the corner followed by the downstream acceleration. Previous studies [8–10] demonstrated that the lift coefficient increases linearly with the deflection angle. 

Chung [11] demonstrated that *M* and *η* are the two major parameters affecting the type of flow field in compressible convex-corner flows. The hypersonic similarity parameter (*Mη*) [12] and a similar combined supersonic-hypersonic similarity parameter $\left(\sqrt{1-M^{2}}\eta \right)$ [13] were examined for scaling the expansion flows. However, the parameter *M* ^2^ *η* appears to be more suitable to characterize the flow characteristics, including peak Mach number, interaction region, and amplitude of peak pressure fluctuations [11, 14]. Further, according to the hypothesis that small streamline deflections produce proportionally small change in Mach number and pressure, a hodograph solution for compressible flow past a corner was given by Verhoff et al. [15]. Another flow similarity parameter, $M^{2}\eta /\sqrt{1-M^{2}}$, was employed by Chung et al. [16] to categorize flow regimes of compressible convex-corner flows. In compressible concave-corner flows, Chung [17] demonstrated that the characteristics of the flow can be scaled with *Mη*, in which stronger upstream compression and downstream expansion are observed with increasing *M* and *η*. Note that there are only slight variations in surface pressure fluctuation coefficients. 

Small perturbation theories have been applied to a number of aeronautical problems, in which the flow is characterized by a small deviation of the flow from its original uniform flow. Linearized solutions are useful for explicitly identifying trends and governing parameters. Consider a slender body at hypersonic speeds, the surface pressure coefficient can be written in terms of hypersonic similarity parameter (*K*) and specific heat ratio (*γ*), in which [13]

(1) Cp=2η2γK2[(1−γ−12K)2γ/(γ−1)−1]

or

(2) Cpη2=f(K,γ).

As mentioned above, the parameters $M^{2}\eta /\sqrt{1-M^{2}}$ and *Mη* were employed to characterize compressible convex- and concave-corner flows, respectively [16]. A common similarity parameter for subsonic corner flows is of interest. Therefore, the present study adopts a combined parameter $K^{*}(=M^{2}/\eta \sqrt{1-M^{2}})$ as a governing parameter to scale the flow characteristics in both subsonic convex- and concave-corner flows for rapid estimation of the interaction region, peak Mach number, and peak pressure fluctuations.

## 2. Experimental Setup

### 2.1. Transonic Wind Tunnel

The experiments were conducted at a transonic wind tunnel of blowdown type, located at Aerospace Science and Technology Research Center, National Cheng Kung University, Taiwan. Major components of the facility include compressors, air dryers, cooling water system, storage tanks, and the tunnel. The dew point of high-pressure air through the air dryers is maintained at −40°C under normal operation conditions. Operating Mach number ranges from 0.2 to 1.4, and simulated Reynolds number is up to 20 million per meter. The test section is 600 × 600 mm and 1500 mm long. In the present study, the test section was assembled with solid sidewalls and perforated top/bottom walls. The freestream Mach numbers were 0.33 and 0.64 ± 0.01, and the stagnation pressure and temperature were 172 ± 0.5 kPa and room temperature, respectively. For the data acquisition system, the LeCroy waveform recorders were used. A host computer with CATALYST software controlled the setup of LeCroy waveform recorders through a LeCroy 8901A interface. All input channels were triggered simultaneously. 

### 2.2. Test Model

**Figure 1:**
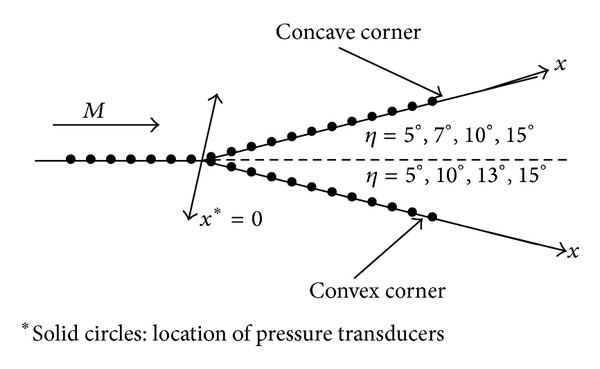
Experimental setup.

The test model consisted of a flat plate and an interchangeable instrumentation plate. The test model was 150 mm wide and 600 mm long, which was supported by a single sting mounted on the bottom wall of the test section, as shown in Figure 1. The concave corner with 5-, 7-, 10-, and 15-deg angles or the convex corner with 5-, 10-, 13-, and 15-deg angles was located at 500 mm from the leading edge of the flat plate. Along the centerline of each instrumentation plate, 19 pressure taps (6 mm apart and 2.5 mm in diameter) were drilled perpendicular to the test surface. All the pressure transducers were flush-mounted to the test surface. The side fences of the instrumentation plate were also installed to prevent cross-flow. A study by Miau et al. [18] indicated that the transition of the boundary layer under the present test condition is close to the leading edge of the flat plate, indicating a turbulent boundary layer at the measurement locations. The boundary layer thickness, *δ*, at 25 mm upstream of the corner was approximately 7.0 mm. 

### 2.3. Experimental Techniques

The Kulite (Model XCS-093-25A, B screen) pressure transducers, which were powered by a TES Model 6102 power supply at 15.0 volts, were employed for pressure measurements. Their outer diameter and sensing element are 2.36 mm and 0.97 mm, respectively. Note that the perforated screen of the pressure transducers might limit the frequency response to only 50 kHz [19]. To improve the signal-to-noise ratio, external amplifiers (Ecreon Model E713) were also employed. With a gain of 20, the roll-off frequency is about 140 kHz. The typical sampling period is 5 *μ*s (200 kHz). Each data record possesses 131,072 data points for statistical analysis. The data were divided into 32 blocks. The mean values of each block (4,096 data points) were calculated. Variations of the blocks are estimated to be 0.43 and 0.13 percent for the mean surface pressure coefficient, *C*_*p*_, and the fluctuating pressure coefficient, *C*_*σp*_, respectively, which were taken as uncertainty of the experimental data.

## 3. Results and Discussions

### 3.1. Mean and Fluctuating Surface Pressure Distributions

**Figure 2:**
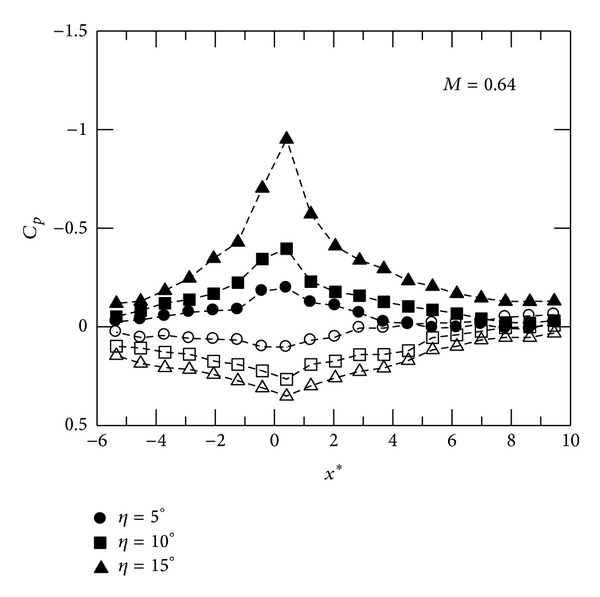
Distributions of pressure coefficient, *M* = 0.64. Hallow symbol: concave corner. Solid symbol: convex corner.

**Figure 3:**
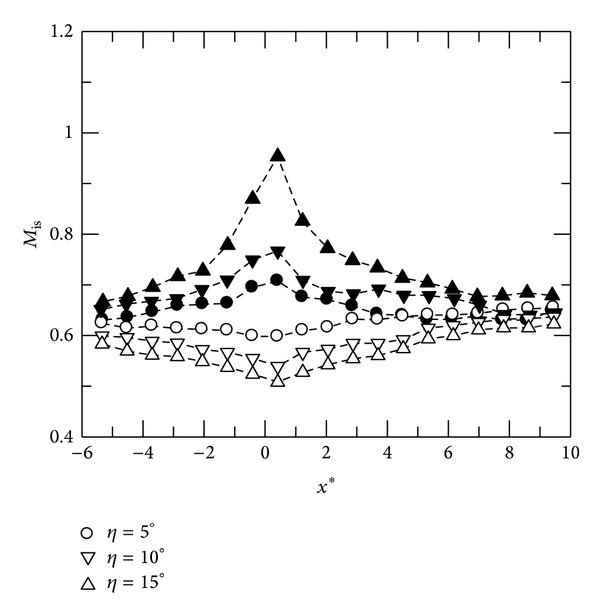
Mach number distributions, *M* = 0.64. Hallow symbol: concave corner. Solid symbol: convex corner.

Distributions of the mean surface pressure coefficient *C*_*p*_ at *M* = 0.64 are shown in Figure 2, where x*( = *x*/*δ*) is the normalized streamwise distance. The origin of the *x* coordinate is set at the corner. The solid symbol corresponds to convex-corner flows, while the hollow symbol denotes concave-corner flows. It is known that viscous-inviscid interactions in subsonic corner flows affect displacement thickness (or effective local wall surface) near the corner apex [7]. Thus, as can be seen, convex-corner flows accelerate gradually upstream of the corner followed by stronger expansion and then downstream compression. The minimum pressure coefficient is observed near the corner apex. There are stronger upstream expansion and downstream recompression at *η* = 15°. It is also noted that the level of *C*_*p*_ tends to an equilibrium value at further downstream locations and decreases when *η* increases. For concave-corner flows at *M* = 0.64, the pressure distributions show a similar shape. The flow decelerates upstream of the corner followed by expansion. The amplitude of *C*_*p*_ appears to increase and decrease linearly within the upstream and downstream influence regions. At further downstream locations, there is a more positive *C*_*p*_ at *η* = 15°. Moreover, the interaction region tends to expand in both upstream and downstream directions for both test cases, indicating stronger viscous-viscid interactions when *η* increases. It is also noted that both upstream and downstream pressure gradients are less significant for convex-corner flows. Further, Mach number distributions at *M* = 0.64 are shown in Figure 3. For concave-corner flows, mild variations in streamwise Mach number are observed. The flow expands suddenly and reaches a peak Mach number, *M*_*p*_, near the convex corner, approaching sonic condition at *η* = 15°. 

**Figure 4:**
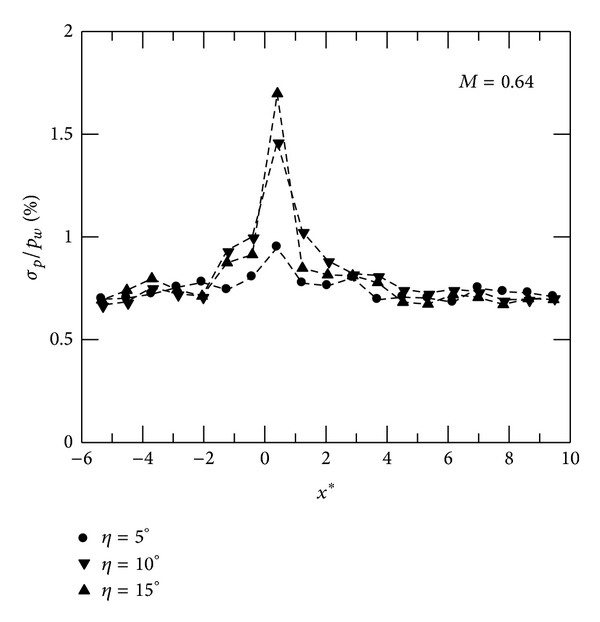
Distributions of surface pressure fluctuations, *M* = 0.64.

The distributions of normalized surface pressure fluctuations for convex-corner flows at *M* = 0.64 are shown in Figure 4. *σ*_*p*_/*p*_*w*_ corresponds to the local variation of surface pressure fluctuations. It can be seen that *σ*_*p*_/*p*_*w*_ increases upstream of the corner and reaches the maximum at immediately downstream location. The rise in *σ*_*p*_/*p*_*w*_ corresponds to the initial pressure rise of mean surface pressure or downstream adverse pressure gradient, as shown in Figure 2. The value of peak pressure fluctuations is higher when *η* increases. At further downstream locations, the amplitude of *σ*_*p*_/*p*_*w*_ approaches an equilibrium level. Note that there are minor variations in *σ*_*p*_/*p*_*w*_ for subsonic flow over a concave corner.

### 3.2. Flow Similarity

**Figure 5:**
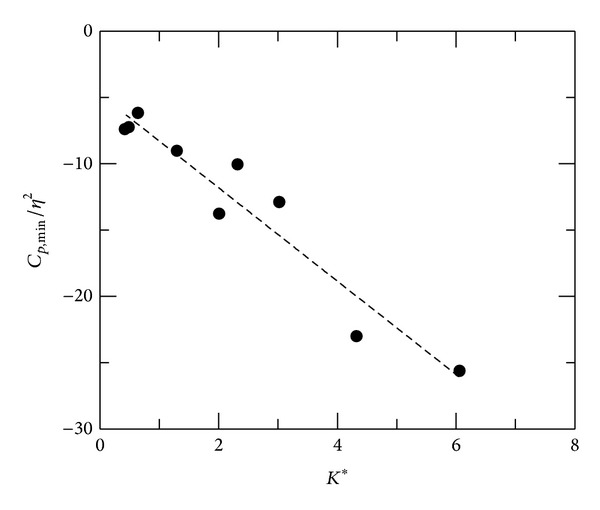
Minimum pressure coefficient, convex-corner flows.

**Figure 6:**
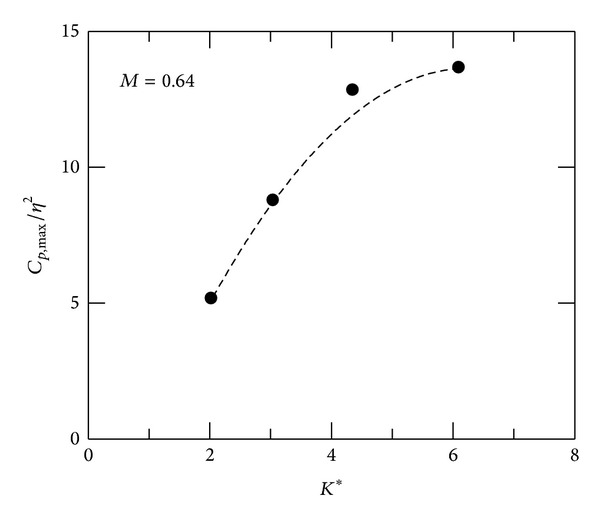
Maximum pressure coefficient, concave-corner flows.

**Figure 7:**
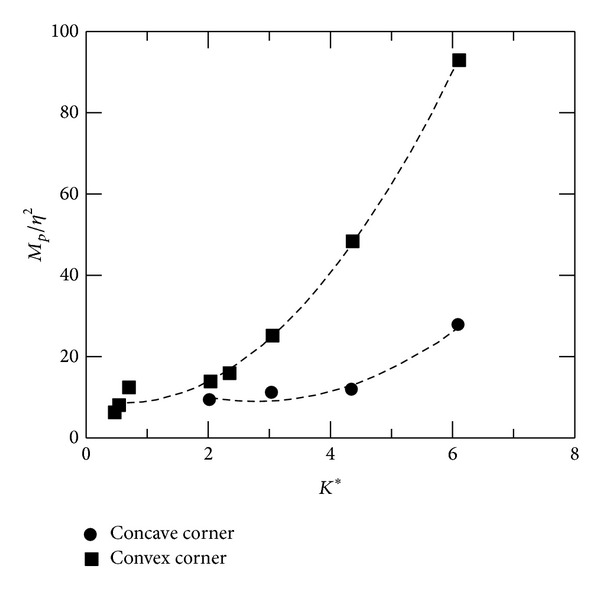
Peak Mach number.

The presence of a convex corner in a subsonic uniform flow results in expansion near the corner, corresponding to the increment in displacement thickness by viscous-inviscid interaction. The minimum pressure coefficients *C*_*p*,min⁡_ are associated with freestream Mach number and deflection angle. In Figure 5, *C*_*p*,min⁡_/*η* ^2^ is plotted against K* for all test cases. It is noted that *η* is in radian in the following analyses. Although the data are slightly scattered, it can be seen that *C*_*p*,min⁡_/*η* ^2^ decreases linearly with K* in convex-corner flows. For concave-corner flows at *M* = 0.64, there is mild adverse pressure gradient upstream of the corner. *C*_*p*,max⁡_/*η* ^2^ can also be scaled with K*, as shown in Figure 6, in which *C*_*p*,max⁡_/*η* ^2^ increases linearly with K*, but not for the test case of K* = 6.1. *C*_*p*,max⁡_/*η* ^2^ appears to be a quadratic function of K* for concave-corner flows. To elaborate further the correlation of flow expansion/compression near the corner with K*, *M*_*p*_^2^/*η* ^2^ is plotted against K* for all test cases. As shown in Figure 7, *M*_*p*_^2^/*η* ^2^ is a quadratic function of K* for both test cases. *M*_*p*_^2^/*η* ^2^ increases significantly when K* increases. However, there are only mild variations in *M*_*p*_^2^/*η* ^2^ with K* for concave-corner flows, demonstrating more significant viscous-inviscid interactions in subsonic convex-corner flow than in subsonic concave-corner flows.

**Figure 8:**
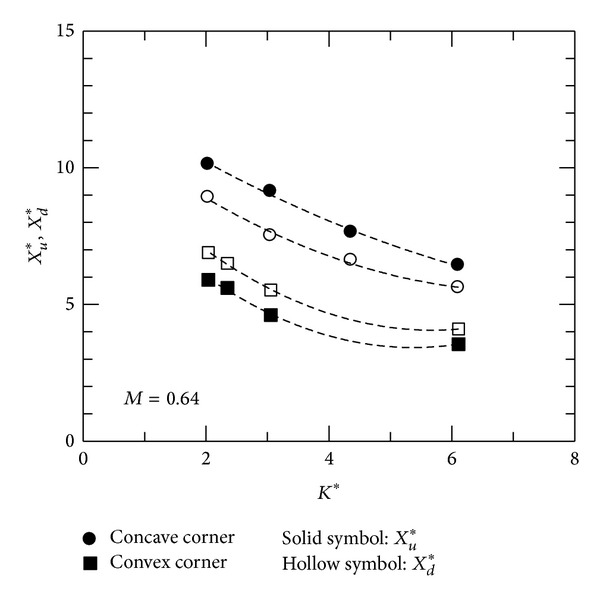
Upstream and downstream influences.

Interaction region, including upstream influence *X*_*u*_ and downstream influence *X*_*d*_, can be employed to highlight viscous-viscid interactions in subsonic convex- and concave-corner flows. Upstream influence, *X*_u_*( = *X*_*u*_/*δ*), can be determined as the intercept of the tangent to the maximum pressure gradient with the undisturbed surface pressure (or *C*_*p*_ = 0). Downstream influence, *X*_d_*( = *X*_*d*_/*δ*), represents the distance for a disturbed boundary layer back to an equilibrium state and can be estimated from the peak pressure near the corner to the intersection of the tangent through the downstream pressure data with the approximately equilibrium downstream pressure [20]. At *M* = 0.64, variations of *X*_u_* and *X*_d_* with K* are shown in Figure 8. As can be seen, *X*_u_* and *X*_d_* decrease linearly with K*for concave-corner flows. For convex-corner flows, there is a shorter interaction region, corresponding to higher upstream and downstream pressure gradients near the corner. *X*_u_* and *X*_d_* appear to be a quadratic function of K*.

**Figure 9:**
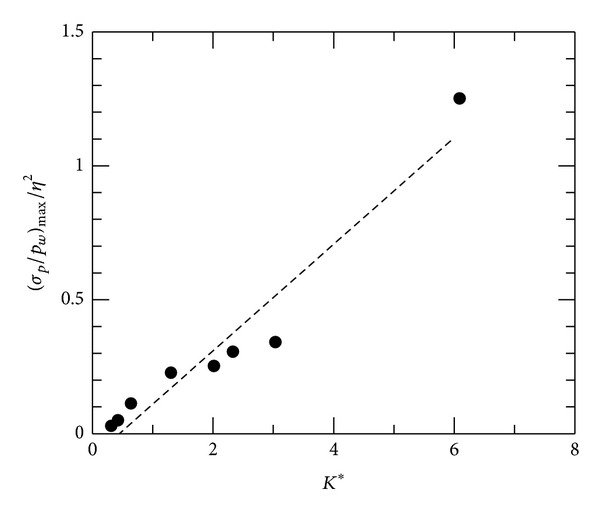
Peak pressure fluctuations, convex-corner flows.

Pressure fluctuations are coupled with global flow unsteadiness. In general, the shear layer structures in the buffer region are responsible for the generation of high-amplitude wall pressure peaks [21]. Laganelli et al. [22] examined wall pressure fluctuations in the attached boundary layer flow. They noted that *σ*_*p*_/*p*_*w*_ is proportional to *M* ^2^. In Figure 9, peak pressure fluctuations, (*σ*_*p*_/*p*_*w*_)_max⁡_/*η* ^2^, are plotted against K* for convex-corner flows. Note that there is only slight variation in surface pressure fluctuations for concave-corner flows. It can be seen that (*σ*_*p*_/*p*_*w*_)_max⁡_/*η* ^2^ increases linearly with K*. More intense pressure fluctuations are associated with higher peak Mach number or flow expansion near the convex-corner apex.

## 4. Conclusions

This paper investigates the flow characteristics of subsonic convex- and concave-corner flows. Mean and fluctuating pressures are presented. A combined flow similarity parameter, K*, based on the small perturbation theory, is employed as a governing parameter to identify the trends in flow expansion/compression, interaction region, and peak pressure fluctuations. For convex-corner flows, variations in minimum pressure coefficient and peak pressure fluctuations can be scaled linearly with K*, but not for upstream and downstream influences. Upstream compression in concave-corner flows appears to be a quadratic function of K*. The results imply that there are small streamline deflections near corner apex, producing proportionally small change in Mach number and pressure. The present results can also be used for a quick estimation of aerodynamic characteristics of a deflected surface in subsonic flows.

## Nomenclature

*C*_*p*_: Pressure coefficient, (*p*_*w*_ − *p*_*∞*_)/*q*
*C*_*σ*_*p*__: Fluctuating pressure coefficient, (*σ*_*p*_ − *σ*_*p∞*_)/*q*
*M*: Freestream Mach number
*M*_*p*_: Peak Mach number near corner apex
*p*_*w*_: Mean surface pressure
*p*_*∞*,_: Freestream mean static pressure
*q*: Freestream dynamic pressure
*x*: Coordinate along the surface of the corner, cm
*x**: Normalized streamwise distance, *x*/*δ*
*X*_*u*_*: Upstream influence, *X*_*u*_/*δ*
*X*_*d*_*: Downstream influence, *X*_*d*_/*δ*
*K**: Similarity parameter, $M^{2}/\eta \sqrt{1-M^{2}}$
*η*: Corner angle
*δ*: Incoming boundary layer thickness, mm
*σ*_*p*_: Standard deviation of surface pressure.

## References

[1] Dolling DS, Or CT (1985). Unsteadiness of the shock wave structure in attached and separated compression ramp flows. *Experiments in Fluids*.

[2] Dolling DS (2001). Fifty years of shock-wave/boundary-layer interaction research: what next?. *AIAA Journal*.

[3] Adamsom TC (1967). Effect of transport properties on supersonic expansion around a corner. *Physics of Fluids*.

[4] Chung K-M, Lu FK (1993). Damping of surface pressure fluctuations in hypersonic turbulent flow past expansion corners. *AIAA Journal*.

[5] Bolonkin A, Gilyard GB Estimated benefits of variable-geometry wing camber control for transport aircraft.

[6] Szodruch J, Hilbig R (1988). Variable wing camber for transport aircraft. *Progress in Aerospace Sciences*.

[7] Ruban AI, Wu X, Pereira RMS (2006). Viscous-inviscid interaction in transonic Prandtl-Meyer flow. *Journal of Fluid Mechanics*.

[8] Smith FT, Merkin JH (1982). Triple-deck solutions for subsonic flow past hymps, steps, concave or convex corners and wedged trailing edges. *Computers and Fluids*.

[9] Chung K-M (2004). Aerodynamic characteristics of deflected surfaces in compressible flows. *Journal of Aircraft*.

[10] Chung K-M (2006). Investigation on compressible ramp flows. *Journal of Aeronautics, Astronautics and Aviation. Series A*.

[11] Chung K-M (2000). Transition of subsonic and transonic expansion-corner flows. *Journal of Aircraft*.

[12] Anderson JD (1990). *Moden Compressible Flow: With Historical Perspecive*.

[13] van Dyke MD (1951). The combined supersonic and hypersonic similarity rule. *Journal of Aeronautical Science*.

[14] Chung K-M (2002). Investigation on transonic convex-corner flows. *Journal of Aircraft*.

[15] Verhoff A, Stockesberry D, Michal T Hodograph solution for compressible flow past a corner and comparison with Euler numerical predictions.

[16] Chung K-M, Chang P-H, Chang K-C (2012). Flow similarity in compressible convex-corner flows. *AIAA Journal*.

[17] Chung K-M (2003). Characteristics of compressible concave-corner flows. *Journal of Aircraft*.

[18] Miau JJ, Cheng J, Chung KM, Chou JF The effect of surface roughness on the boundary layer transition.

[19] Gramann RA, Dolling DS (1990). Detection of turbulent boundary-layer separation using fluctuating wall pressure signals. *AIAA Journal*.

[20] Chung K (1998). Interaction region of turbulent expansion-corner flow. *AIAA Journal*.

[21] Kim J, Kim K, Sung HJ (2003). Wall pressure fluctuations in a turbulent boundary layer after blowing or suction. *AIAA Journal*.

[22] Laganelli AL, Martellucci A, Shaw LL (1983). Wall pressure fluctuations in attached boundary-layer flow. *AIAA Journal*.

